# Women Know Better What Other Women Think and Feel: Gender Effects on Mindreading across the Adult Life Span

**DOI:** 10.3389/fpsyg.2017.01324

**Published:** 2017-08-02

**Authors:** Renata Wacker, Sven Bölte, Isabel Dziobek

**Affiliations:** ^1^Department of Education and Psychology, Freie Universität Berlin Berlin, Germany; ^2^Berlin School of Mind and Brain, Humboldt-Universität zu Berlin Berlin, Germany; ^3^Center of Neurodevelopmental Disorders, Pediatric Neuropsychiatry Unit, Department of Women’s and Children’s Health, Karolinska Institutet Stockholm, Sweden

**Keywords:** social cognition, mindreading, emotion recognition, own-gender bias, gender differences, age differences

## Abstract

Research recurrently shows that females perform better than males on various mindreading tasks. The present study contributes to this growing body of literature by being the first to demonstrate a female own-gender mindreading bias using a naturalistic social cognition paradigm including female and male targets. We found that women performed better at reading others’ minds, and that they were specifically more capable to read female targets, an own-gender target effect absent in men. Furthermore, a non-linear negative effect of perceiver age on mindreading performance was examined within a sample covering the age range of 17–70 years, as indicated by a stronger performance decrease setting on by the age of 30 years and continuing throughout middle and old age. These findings add to a more comprehensive understanding of the contextual factors influencing mindreading performance in typically developing adults.

## Introduction

Much of social interaction occurs “in silence,” as people exchange information about emotions, thoughts, and intentions in implicit ways. Thus, everyday social life requires us to hypothesize based on our interaction partners’ non-verbal expressions and behaviors, implicit or ambiguous verbal messages, and actions conveying thoughts and intentions indirectly. This social-cognitive capacity to infer others’ emotional, cognitive, or motivational mental states is referred to as mindreading. It relies on various visual, verbal and symbolic cues (e.g., facial expressions, voice, gestures, and body movements) occurring in social interaction. Thus, the abilities serving this capacity range from emotion recognition and empathic accuracy to attributing intentions, inferring thoughts, understanding faux pas and false beliefs. Social psychological research increasingly recognizes the crucial role of contextual factors, e.g., perceiver characteristics or perceiver-target interactions, for a better understanding of the interpersonal functions of mindreading beyond its intrapersonal mechanisms and neural correlates (Thomas and Fletcher, 2003; Zaki and Ochsner, 2011).

Research recurrently demonstrates that females perform better on various mindreading tasks such as mindreading accuracy (Thomas and Fletcher, 2003), mental state inference (Kirkland et al., 2013), faux pas understanding (Ahmed and Miller, 2011), facial expression processing (McClure, 2000), or emotion labeling (Montagne et al., 2005). Gender differences in mindreading have been linked to biological sex differences. Although this account is not uncontroversial (Valla et al., 2010), Baron-Cohen (2002) proposes that the “typical female” brain would engage more strongly in understanding mental states of (social) agents, whereas the “typical male brain” tends to analyze non-agentic systems. Connellan et al. (2000) actually observed sex-specific stimuli preferences in newborns (face in females vs. moving mobile in males). This study is the only one demonstrating such early sex differences using these two objects. However, it has also been found that female newborns have a stronger interest in eye contact as compared to male newborns (Hittelman and Dickes, 1979). These early sex differences are assumed to initiate sex-specific integration of brain systems, which develop as differences in social perception and cognition (McGuinness and Pribram, 1979; Byrd-Craven and Geary, 2013). Differences in interest also seem to exist in adult life since women prefer to work with people, and men with things [Cohen’s *d* = 0.9 in the meta-analysis by Su et al. (2009)].

It has been debated whether women have a higher ability or “just” a stronger motivation to read others’ minds. Since this controversy started (Ickes et al., 2000), the performance enhancing effects of the specifically female gender role motivation to be an accurate mindreader have been examined (e.g., Thomas and Maio, 2008). Hodges et al. (2011, p. 59) conclude that despite higher mindreading motivation “women probably hold a slight general edge over men” given the consistently found female advantage even in the absence of motivational factors. They further relate it to some specific features of the *female* standard stimulus person used in these studies, speculating that a female target might appeal stronger to women than to men. Unfortunately, this line of research did not consider the possible interaction of perceiver gender and target gender systematically. In face recognition literature, however, a bias for own-gender targets in women but not in men has been consistently reported (Lewin and Herlitz, 2002; Herlitz and Lovén, 2013). Herlitz and Lovén (2013) provide a developmental explanation by arguing that female newborns’ stronger interest for faces, and the fact that their primary caregivers are women, result in perceptual expertise for female faces based on mutual reinforcement of preference and familiarity over time. Females’ stronger tendency to establish more intimate “face-to-face” same-gender friendships (as opposed to males’ activity oriented “side-by-side” friendships) might further strengthen their same-gender face recognition bias (Rehnman and Herlitz, 2007). Face perception is considered as one core mechanism of social cognition since Schultz et al. (2003) had provided evidence for the notion that the fusiform face area, a region specialized for face perception, also represents semantic information about “peopleness” (personal agency), and is thus involved in attributing mental states to objects. This basic idea, i.e., that recognizing people’s faces is linked to reading their mental states, combined with the mentioned developmental explanation for the own-gender face recognition bias in women leads to the question whether a specifically female own-gender mindreading bias actually exists. We argue that females’ perceptual expertise for female faces presumably also facilitates their social-cognitive expertise for female minds.

Age is another perceiver variable affecting the ability to understand other people’s mental states. While earlier evidence had suggested a positive effect of age on mindreading as a manifestation of increasing “social wisdom” (Happé et al., 1998), a more recent meta-analytic review clearly demonstrated that younger adults perform better on mindreading tasks than older adults across various domains (affective/cognitive/mixed) and modalities (verbal/visual, static/dynamic; Henry et al., 2013). Yet, most of these findings rely upon performance differences between extreme age groups, not continuous age data. Therefore, a comprehensive picture of adult mindreading performance in women and men across the whole adult lifespan is still lacking. An exception is evidence derived from a sample of >70,000 adults (18–90 years) indicating an inverted u-shaped relationship of age and self-reported perspective taking with a performance peak at 50–60 years (O’Brien et al., 2012). This hints toward a positive relationship between age and mindreading until mature adulthood, and a negative relationship in old age. Though perspective taking captures only one aspect of the ability to read other people’s minds, this result does not fit well with other literature on aging and mindreading as younger adult groups have been almost consistently found to perform better on mindreading tasks as compared to any older age group. However, the finding reported by O’Brien et al. (2012) suggests a possible non-linear relationship between age and mindreading but the exact nature of this relationship has yet to be investigated on the basis of a more objective task measuring various components of mindreading.

In the present study, we examined effects of perceiver gender and age, and target gender on mindreading performance as assessed with an ecologically valid test that captures the broad composite of everyday mindreading targeting multiple characters of both genders. We hypothesized (i) a perceiver gender effect on mindreading, i.e., women perform better than men; (ii) a specifically female own-gender mindreading bias, i.e., women read female targets more accurately than male targets; and (iii) a negative non-linear relationship of perceiver age and mindreading.

## Materials and Methods

### Participants

The presented data are based on a multi-site data collection comprising 14 studies conducted in Germany (e.g., Preißler et al., 2010; Montag et al., 2011; Ritter et al., 2011; Buhlmann et al., 2015). Only data from typically developing participants were used for the current analyses while individuals with documented clinical diagnosis were excluded. The resulting total sample (*N* = 545) comprised of 304 females (56%) and 241 males (44%). This large and statistically powerful sample of convenience was used with no *a priori* sample size calculation. Participants’ average age was *M* = 31.93 years (*SD* = 11.42; range: 17.62–70.00), and their mean duration of education was *M* = 13.49 years (*SD* = 2.67; range: 9.00–20.00).^1^ Females were significantly older (*M* = 32.95, *SD* = 12.15) than males (*M* = 30.63, *SD* = 10.32; *p* = 0.16). No gender difference was present for education duration. The studies were approved by the respective local ethics committees, e.g., Ethics Committee of Charité – Universitätsmedizin Berlin. Two of the 14 studies included typically developing individuals only and, thus, did not legally require ethics approvals for collecting behavioral data with our measure. In addition, all subjects gave written informed consent in accordance with the Declaration of Helsinki.

### Measure

Mindreading was measured with The Movie for the Assessment of Social Cognition (MASC; Dziobek et al., 2006). The MASC is an explicit mindreading performance test based on a narrative fictional film providing naturalistic verbal and non-verbal stimuli of dynamic social interaction. It captures affective and cognitive mental state inference, such as Theory of Mind, emotion recognition and perspective taking. It includes four targets who exhibit the full variety of verbal and non-verbal information, and express their emotions, thoughts and intentions in dynamic interaction. The 15-min movie is about two female and two male middle aged adults preparing and getting together for dinner, and focuses on their social communication and interaction. The movie is stopped 45 times in order to inquire about the characters’ thoughts, intentions or emotions (e.g., “What is Cliff thinking?,” “Why is Betty saying this?,” “What is Michael feeling?”). The response format of the current MASC version is a multiple-choice structure with one correct response and three distractors for each of the 45 questions. The possible total score ranges from 0 to 45, and the subscore for female/male targets from 0 to 100%. Subscores represent the percentage of correctly answered items (based on 26 items targeting female characters, and 18 items targeting male characters; one item targets 3 characters at once, and thus is not included). The MASC was administered with Microsoft Office PowerPoint or Presentation. In the original validation study (Dziobek et al., 2006) high correlations of the MASC score with social functioning were found in individuals on the autism spectrum, and the test has been shown to have high test-retest reliability (ICC = 0.97). For further details with regard to test development, stimuli and administration see Dziobek et al. (2006).

We assessed the psychometric properties of the MASC based on classical item analysis and confirmatory factor analysis. A larger sample of *N* = 713 with 56% female and 44% male participants was available for this purpose.^2^ Participant’s average age was *M* = 30.80 years (*SD* = 11.62; range: 12.61–70.00) and their mean duration of education was *M* = 14.02 years (*SD* = 2.26; range: 9.00–20.00).^3^ The item analysis (item difficulties, item-total correlations) and reliability analysis in terms of internal consistency (Cronbach’s alpha) were performed using SPSS 21.0, and the confirmatory factor analysis for categorical data was conducted with Mplus 6.1 (Muthén and Muthén, 2010-2012). McDonald’s (1999) omega was computed based on the estimated item-loadings.

The average MASC total score was *M* = 34.15 (*SD* = 5.25; *Mdn* = 35.00) and ranged from 9 to 45. The item difficulty as represented by item mean ranged from *M* = 0.52 (Item 35) to *M* = 0.94 (Item 11 and 45). The item-total correlations ranged from r_it_ = 0.08 (Item 13) to r_it_ = 0.37 (Item 11 and 28). To assess the assumption of unidimensionality the confirmatory factor analysis was performed with only one latent factor using the WMSLV estimation method. The RMSEA indicated good model fit, but the CFI and TLI were below the threshold for acceptability of 0.95 [χ^2^(945) = 1261.08, *p* < 0.05; CFI = 0.83; TLI = 0.82; RMSEA = 0.022]. The discrepancy between the CFI, TLI and the RMSEA could be explained by the relatively low average tetrachoric correlation between the items. The factor loadings of the unidimensional model ranged from 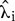 = 0.16 (Item 04) to 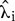 = 0.71 (Item 11), indicating that item 04 has the lowest and item 11 the highest association with the latent factor. The estimated communalities ranged from 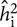 = 0.02 (Item 13) to 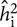 = 0.50 (Item 11), referring to the relative proportion of the latent response variable’s variance that is explained by the factor. The threshold parameters ranged from 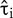 = -1.59 (Item 11) to 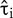 = -0.07 (Item 35), which means that item 11 is the easiest and item 35 the most difficult item to solve. Cronbach’s α based on the classical item analysis was 0.74, and Mc Donald’s ω was 0.88. The results of the classical item analysis and confirmatory factor analysis are displayed in Appendix (see Supplementary Material), allowing for a detailed overview of the items’ psychometric properties.

The MASC is widely used in clinical studies as a sensitive test of mindreading deficits in, e.g., autism, borderline personality, and body dismorphic disorders (Dziobek et al., 2006; Preißler et al., 2010; Buhlmann et al., 2015), and also recognized as a suitable measure of individual differences in typically developing adults (Turner and Felisberti, 2017). Examination of the psychometric properties in the present study’s sample demonstrated that the MASC is an internally consistent, unidimensional test of medium difficulty well-suited to assess individual differences in mindreading.

### Statistical Analyses

To assess the relationships between perceiver gender, perceiver age, and mindreading performance, we conducted a multiple robust regression of MASC total score on age (centered) and gender (dummy coded, 0 = female, 1 = male), using the package “robustbase” (Hlavac, 2015) of the software R 3.1.0 (R Core Team, 2013) due to its robustness against outliers.^4^ We also included the quadratic term of age (centered) in order to examine its non-linear effect. Secondly, we performed a 2 × 2 repeated-measures ANCOVA in SPSS to examine the interaction effect of perceiver gender (between-subject factor) and target gender (within-subject factor) on MASC subscores (age and age squared were both centered and entered as covariates). *Post hoc* within- and between-group comparisons were Bonferroni corrected, and group differences were compared by effect size as measured by partial eta squared.

## Results

### Effects of Perceiver Gender and Perceiver Age on Mindreading Performance

**FIGURE 1:**
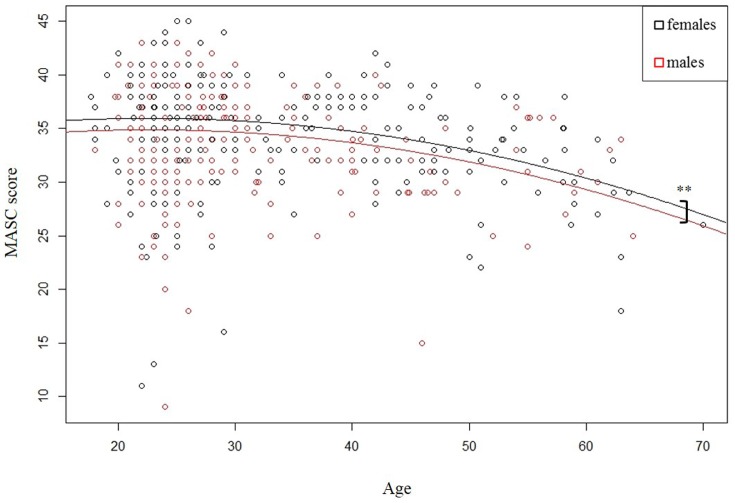
Non-linear relationship between age and mindreading performance for females and males.

Participants’ predicted MASC score was equal to 35.609 – 1.067 (gender) – 0.074 (age) – 0.004 (age^2^), *R*^2^ = 0.121. Gender predicted mindreading performance with males scoring lower on the MASC than females (*p* = 0.005). Age was a negative predictor (*p* = 0.002), and the quadratic term of age was also significant (*p* = 0.005), indicating a non-linear relationship of age and mindreading during adulthood. As illustrated by **Figure 1**, the decrease of MASC score was more pronounced in middle and old adult age than in late adolescence and young adulthood (<30 years). There was no evidence for a significant age × gender interaction effect.

### Interaction Effect of Perceiver Gender and Target Gender on Mindreading Performance

**FIGURE 2:**
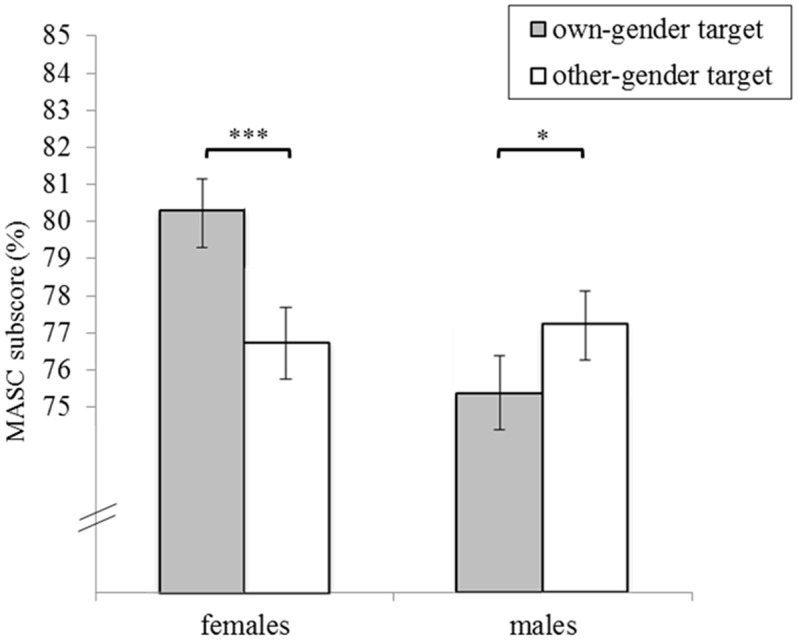
Interaction effect of perceiver and target gender on mindreading performance. Age (covariate) is evaluated at the level of 31.93 years. Error bars represent ± 1 SE. ^∗^*p* < 0.05, ^∗∗∗^*p* < 0.001.

We found a trend for an interaction effect of perceiver and target gender on mindreading performance, *F*(1,541) = 2.81, *p* = 0.09, η^2^ = 0.005, 95% CI (0.000, 0.024). *Post hoc* within-group comparisons (**Figure 2**) revealed that subscores for own-gender targets (*M* = 80.34, *SE* = 0.84) were significantly higher as compared to other-gender targets (*M* = 76.76, *SE* = 0.94) in female participants, *F*(1,541) = 17.84, *p* < 0.001, η^2^ = 0.032, 95% CI (0.009, 0.066), whereas the reverse pattern occurred in male participants (own-gender targets: *M* = 75.36, *SE* = 0.99; other-gender targets: *M* = 77.26, *SE* = 0.88; *F*[1,541] = 4.53, *p* = 0.03, η^2^ = 0.008, 95% CI [0.000, 0.030]). *Post hoc* between-group comparisons indicated that females’ subscore for female targets was significantly higher than males’ subscore for female targets, *F*(1,541) = 9.66, *p* = 0.002, η^2^ = 0.018, 95% CI (0.002, 0.045), and that subscores for male targets did not differ significantly between females and males, *F*(1,541) = 1.57, *p* = 0.211, η^2^ = 0.003, 95% CI (0.000, 0.019). In addition to the interaction effect, the main effect of target gender was significant with higher mindreading performance for female targets (*M* = 77.20, *SE* = 0.49) than male targets (*M* = 75.21, *SE* = 0.55), *F*(1,541) = 14.89, *p* < 0.001, η^2^ = 0.027, 95% CI (0.007, 0.059).

## Discussion

We provide evidence that women are better able than men to infer other women’s mental states. This result specifies the understanding of gender effects which have been reported by previous research showing that women hold an advantage over men across various components of mindreading (McClure, 2000; Thomas and Fletcher, 2003; Montagne et al., 2005; Ahmed and Miller, 2011; Kirkland et al., 2013). In the present study, women outperformed men particularly when asked to read female targets, whereas no such own-gender bias was found in men. To the best of our knowledge, this is the first evidence to demonstrate the specifically female own-gender mindreading bias. It parallels the gender effects repeatedly found in face recognition literature showing that women have a stronger advantage in recognizing female faces (Herlitz and Lovén, 2013). How could females’ perceptual expertise for female faces have developed into a social-cognitive expertise for female minds? First, we assume an equal-mindreaders effect. Apart from women’s interest for and familiarity with other females, their bias might be further reinforced by higher reciprocity in interactions with females possessing equal social-cognitive skills as compared to male mindreaders with relatively lower skills, and thus draw females more toward equal interaction partners throughout their social-cognitive development. Additionally, positive relationship outcomes of mindreading and perspective taking, such as greater intimacy and closeness (Chow et al., 2013), might further reinforce reciprocal mindreading between females, since women seek talking and emotion sharing in same-gender friendships more than men (Caldwell and Peplau, 1982). Another developmental explanation is a superior-mindreader effect, which could manifest the female own-gender bias especially during adolescence. This period’s most important developmental task is identity formation (Erikson, 1968; Kroger et al., 2010). Possibly, seeking self-understanding motivates young females to engage in astute social communication with older and “wiser” women, since the recursive nature of social-cognitive inferences (“I think that she thinks that I believe…”) allows for learning about one’s own thoughts and feelings through the reflection of the self in the mind of another. Given that women are better mindreaders than men, adolescents might prefer them for the sake of better self-understanding. Adolescent girls actually self-disclose more with their mother than father, whereas boys share less with their father than mother (Rivenbark, 1971). This interaction pattern seems to persist beyond adolescence since women generally disclose more than men toward same-gender targets (Dindia and Allen, 1992). Finally, the female own-gender mindreading bias could be also explained by the fact that women are more stimulating as a target of mindreading. They have shown to be more emotionally expressive than men (Gross and John, 1995), and therefore might provide richer input and a stronger appeal for others to read them. This could also account for men’s higher performance in reading female targets as compared to male targets in our sample. However, female targets’ expressivity might specifically interact with other womens’ higher mindreading ability, thus resulting in a better understanding of a more expressive target by a more astute perceiver.

Taken together, we propose various mechanisms of the specifically female own-gender bias in mindreading: Women are better at understanding other females’ feelings and thoughts because interactions with other women might offer them higher reciprocity of mindreading skills, a realization of their relationship motives (e.g., emotion sharing), self-reflection with a superior mindreader (especially during adolescence), and a more stimulating, emotionally expressive target of mindreading. These putative mechanisms should be further examined in future research to better understand why the female own-gender bias exists.

Furthermore, the non-linear negative effect of age on mindreading performance found in the present study extends the existing literature on age and social cognition by providing a more differentiated picture of mindreading across the adult life span. The vast majority of previous studies relied upon mean differences between extreme age groups and/or lacking groups representing mature adulthood. These designs were not suited to detect non-linear trajectories across the entire adult life span. Our regression analysis using age-continuous data ranging from 17 to 70 years shows the onset of a negative trajectory by the age of approximately 30 years, and continuation throughout middle and old age. A non-linear effect of age on perspective taking was already reported by O’Brien et al. (2012). Their results differed as they found an inverted u-shaped trajectory peaking around 50–60 years. However, perspective taking represents only a very specific component of mindreading which, especially when measured via self-report, might be confounded with a prosocial *motivation* (i.e., willingness to take the perspective of another). Prosociality itself increases with age (Sze et al., 2012). The more objective mindreading test used in the present study is presumably less prone to motivational confounds, and thus, better suited to measure actual performance differences related to age. Our results further complement the literature on social-cognitive aging as they are based on a naturalistic measure assessing the various components of everyday mindreading in a more comprehensive fashion as compared to previous studies, which for instance focused either on Theory of Mind or emotion labeling.

Since we did not use longitudinal data, a cohort effect could have possibly confounded the age effect. In order to explore this we repeated the regression analysis with a MASC total score reduced by seven items which might be biased by specific (lack of) knowledge or social norms probably present in older participants (e.g., traditional view regarding the role of female host). The results, however, did not change which indicates genuinely age-related performance differences. Nonetheless, it should be replicated with longitudinal data to exclude the possibility of cohort effects. Another limitation of our analysis is the missing inclusion of indicators of general cognitive ability. The negative age effect on mindreading appears to be similar to age-related differences in general cognitive performance (Salthouse, 2009). However, previous literature has shown that the negative relationship of age and mindreading is only partly associated with age-related general cognitive impairments such as executive functioning and fluid intelligence (Moran, 2013). Finally, we cannot exclude the possible performance enhancing effect of the overt task demand given that participants are explicitly asked to infer the MASC characters’ mental states. At the same time, the test does not produce ceiling performance effects and is a psychometrically sound measure of the individual differences presented in this study.

## Conclusion

This work contributes to the growing literature on the contextual factors of mindreading such as perceiver and target characteristics. By using a mindreading test that includes female as well as male targets, we demonstrated a specifically female own-gender bias in the ability to understand what others think and feel. The proposed social-cognitive mechanism and developmental factors of this bias have to be examined in following studies.

The negative non-linear age effect on mindreading, marked by age-related performance differences setting on by the age of approximately 30 years, further clarifies how this ability might differ throughout adult life. This finding, however, has to be replicated with longitudinal age data in future research.

Finally, other than many of the previous studies on mindreading (or specific components thereof), the results of the present work rely upon a naturalistic social cognition test that captures the broadness of various mindreading components, and accurately assesses subtle individual differences in typically developing adults. As has been already suggested elsewhere (Turner and Felisberti, 2017), using this kind of measures in mindreading studies contributes to the validity of research findings and their applicability to everyday social life.

## Author Contributions

RW and ID designed the study. RW performed the analysis and wrote the manuscript. ID and SB gave feedback on the analysis and the manuscript.

## Conflict of Interest Statement

The authors declare that the research was conducted in the absence of any commercial or financial relationships that could be construed as a potential conflict of interest. The reviewer AH declared a shared affiliation, though no other collaboration, with one of the authors SB to the handling Editor, who ensured that the process nevertheless met the standards of a fair and objective review.

## Footnotes

1 Information on education was available for *N* = 229.

2 Information on gender was available for *N* = 548.

3 Information on age was available for *N* = 632, and on education for *N* = 354.

4 Seven participants scored lower than 3 standard deviations below the MASC mean score.
