# Supplementary material for: Women Know Better What Other Women Think and Feel: Gender Effects on Mindreading across the Adult Life Span
Source: Front Psychol. 2017 Aug 2;8:1324. doi: 10.3389/fpsyg.2017.01324 (PMC5539187; doi:10.3389/fpsyg.2017.01324)
Supplement: Supplementary file 1 [file Data_Sheet_1.docx]

**Appendix**

Item analysis and confirmatory factor analysis (*N* = 713)

| Item | *M* | $r_{it}$ | $\hat{\lambda}_{i}$ | $\hat{h}_{i}^{2}$ | $\hat{\tau}_{i}$ |
| --- | --- | --- | --- | --- | --- |
| 01 | .83 | .24 | .41 | .17 | -.97 |
| 02 | .83 | .23 | .41 | .16 | -.94 |
| 03 | .60 | .14 | .23 | .05 | -.25 |
| 04 | .59 | .12 | .16 | .03 | -.24 |
| 05 | .81 | .23 | .39 | .15 | -.88 |
| 06 | .73 | .20 | .29 | .09 | -.63 |
| 07 | .81 | **.29** | .49 | .24 | -.89 |
| 08 | .81 | .21 | .37 | .14 | -.88 |
| 09 | .72 | .21 | .33 | .11 | -.57 |
| 10 | .80 | .20 | .31 | .10 | -.83 |
| 11 | .94 | **.34** | **.71** | .50 | -1.59 |
| 12 | .63 | .09 | .17 | .03 | -.32 |
| 13 | .62 | .08 | .13 | .02 | -.32 |
| 14 | .67 | .20 | .30 | .09 | -.45 |
| 15 | .68 | .21 | .35 | .12 | -.47 |
| 16 | .87 | **.29** | **.50** | .25 | -1.11 |
| 17 | .90 | .23 | .43 | .18 | -1.26 |
| 18 | .92 | .22 | .44 | .20 | -1.38 |
| 19 | .74 | .20 | .31 | .10 | -.65 |
| 20 | .91 | **.32** | **.60** | .35 | -1.32 |
| 21 | .55 | .19 | .29 | .08 | -.12 |
| 22 | .70 | .20 | .30 | .09 | -.52 |
| 23 | .79 | **.33** | **.52** | .27 | -.82 |
| 24 | .86 | **.27** | .45 | .21 | -1.07 |
| 25 | .89 | **.28** | **.51** | .26 | -1.22 |
| 26 | .55 | .19 | .31 | .10 | -.13 |
| 27 | .85 | .23 | .41 | .17 | -1.05 |
| 28 | .92 | **.34** | **.66** | .44 | -1.43 |
| 29 | .76 | .18 | .28 | .08 | -0.72 |
| 30 | .79 | .24 | .39 | .15 | -.80 |
| 31 | .65 | .19 | .29 | .09 | -.38 |
| 32 | .65 | **.28** | .44 | .20 | -.40 |
| 33 | .76 | **.29** | **.46** | .21 | -.71 |
| 34 | .83 | .22 | .38 | .14 | -.96 |
| 35 | .53 | .16 | .26 | .07 | -.07 |
| 36 | .92 | **.25** | **.50** | .25 | -1.39 |
| 37 | .60 | .17 | .25 | .06 | -.26 |
| 38 | .85 | **.33** | **.54** | .29 | -1.03 |
| 39 | .83 | .12 | .19 | .04 | -.96 |
| 40 | .63 | .14 | .21 | .04 | -.33 |
| 41 | .84 | **.28** | .45 | .21 | -.99 |
| 42 | .48 | **.24** | .36 | .13 | -.06 |
| 43 | .72 | .10 | .17 | .03 | -.60 |
| 44 | .86 | **.28** | .49 | .24 | -1.07 |
| 45 | .94 | .21 | .44 | .20 | -1.58 |

*Note.* Coding of item responses: 0 = wrong, 1 = right; $r_{it}$ *=* item-total correlation, $\hat{\lambda}_{i}$ = estimated standardized loading, $\hat{h}_{i}^{2}$ = communality, $\hat{\tau}_{i}$= estimated threshold parameter; $r_{it}$ ≥ .25 and and $\hat{h}_{i}^{2}$≥ .50 marked in boldface.
